# Laying the Foundation for a Mesothelioma Patient Registry: Development of Data Collection Tools

**DOI:** 10.3390/ijerph20064950

**Published:** 2023-03-11

**Authors:** Joanna M. Gaitens, Melissa Culligan, Joseph S. Friedberg, Erica Glass, Maxwell Reback, Katherine A. Scilla, Ashutosh Sachdeva, Anthony Atalla, Melissa A. McDiarmid

**Affiliations:** School of Medicine, University of Maryland, Baltimore, MD 21201, USA

**Keywords:** mesothelioma, registry, asbestos exposure

## Abstract

Mesothelioma, a cancer of mesothelial cells that line the chest, lungs, heart, and abdomen, is a relatively rare disease. In the United States, approximately 3000 individuals are diagnosed with mesothelioma annually. The primary risk factor for mesothelioma is occupational asbestos exposure which can occur decades prior to disease development, though in approximately 20% of cases, known asbestos exposure is lacking. While several other countries have developed mesothelioma registries to collect key clinical and exposure data elements to allow better estimation of incidence, prevalence, and risk factors associated with disease development, no national mesothelioma registry exists in the U.S. Therefore, as part of a larger feasibility study, a patient exposure questionnaire and a clinical data collection tool were created using a series of key informant interviews. Findings suggest that risk factor and clinical data collection via an on-line questionnaire is feasible, but specific concerns related to confidentiality, in the context of employer responsibility for exposure in the unique U.S. legal environment, and timing of enrollment must be addressed. Lessons learned from piloting these tools will inform the design and implementation of a mesothelioma registry of national scope.

## 1. Introduction

### 1.1. Mesothelioma in the United States

Mesothelioma is a lethal cancer, arising from mesothelial cells lining the pleural space, peritoneum, pericardium, and tunica vaginalis. Approximately 80% of cases are pleural, which is considered incurable and typically portends a life expectancy in the one-to-two-year range. Approximately 10–15% of cases are peritoneal, with the remainder arising in the pericardium and testes [1]. It is an orphan disease with only several thousand cases per year in the United States. The primary risk factor for mesothelioma is occupational asbestos exposure which can occur decades prior to disease development. Research has shown that workers in specific industries, such as construction [2,3,4], ship building and repair [3,5,6,7], mining [8,9], firefighting [10], and railway [2] industries, have an increased risk of exposure to asbestos. However, in approximately 20% of mesothelioma cases, known occupational exposure to asbestos is lacking [11].

In addition to direct occupational asbestos exposure, indirect exposure may occur in family members of the asbestos worker who brings the asbestos mineral fibers home on clothes and shoes [12,13]. Individuals can also be exposed to asbestos and asbestiform fibers in the environment, either through natural weathering processes or man-made activities, thus increasing their risk of developing mesothelioma. For example, in certain communities, such as Libby, Montana, increased rates of mesothelioma have been found in residents exposed to amphibole asbestos in the environment due to previous mining activities [14,15]. However, overall, due to the limited availability of data, it has been difficult to estimate the number of mesothelioma cases caused by non-occupational exposures [11].

Often by the time mesothelioma is diagnosed, a patient’s prognosis is poor, making early identification and recognition of disease development an important step in preventing disease through increased surveillance of “at risk” populations, as is required by the U.S. Occupational Safety and Health Administration (OSHA) in asbestos-exposed occupational cohorts [16]. Quantifying exposure intensity (dose), duration, and other circumstances raising risk in individuals is often imprecise or unknown, making risk estimates to specific persons or populations difficult. Opportunities for more in-depth history taking, especially in light of the absence of occupational asbestos exposure in approximately 20% of mesothelioma patients, may close the exposure history knowledge gap, identifying new exposure sources and permitting a more refined assessment of disease risk and prevention opportunities at the population level.

### 1.2. Public Health Registries as Surveillance Tools

Public health registries have long played an important role in understanding the causes and natural progression of diseases, historically from infectious agents and, over time, for non-communicable diseases, especially cancers [17]. Recently, the registry concept has also been applied to follow groups of people who do not share a common disease, but rather, a common exposure history. An example here would include the World Trade Center (WTC) Health Registry established by the Agency for Toxic Substances and Disease Registry (ATSDR), part of the U.S. Centers for Disease Control and Prevention (CDC), and the New York City Department of Health [18]. This registry enrolls persons who lived, worked, or went to school near the WTC disaster site and those who assisted in rescue and recovery efforts to assess the impact of the 11 September 2001 event on their physical and mental health [18].

As the name implies, a registry is an organized system for collecting and analyzing data on individuals with shared characteristics from which population inferences can be derived [19]. In addition to collecting demographic and medical information, registries often seek validation of the data by collecting detailed test results (such as a pathology report from a biopsy or a specific blood test result). They can serve as powerful tools for tracking disease trends over time, determining the incidence and prevalence of diseases, identifying groups at high-risk, and estimating health service needs [19].

An additional benefit of a registry is that it allows tracking of the pool of persons identified as being at an increased risk of health harm. This tracking enables subsequent contact of the registered persons by health authorities, who may offer health information, opportunities for study participation, or care recommendations. The course of the registrant’s health may also be followed over time through periodic surveys performed by the registry. In the case of rare diseases, such as mesothelioma, tracking of individuals and collection of standardized data can be especially useful in providing a mechanism to unite patients who are geographically dispersed and providing researchers with a more robust dataset to better characterize disease and treatment outcomes [20].

### 1.3. Mesothelioma Registries

To date, several other countries have implemented mesothelioma registries, including Australia, Belgium, France, Germany, Italy, Japan, South Africa, South Korea, Turkey, and the United Kingdom [21]. Although varying in scope, some registries have incorporated the use of patient questionnaires to capture key exposure-related details related to development of mesothelioma. These registries have permitted better estimates of disease incidence and identification of asbestos exposure sources and other risk factors [22,23,24,25,26,27,28,29], thereby advancing scientific knowledge and allowing for preventive approaches to be implemented.

The U.S. experience differs from these country examples, however, in that, there is no national data collection system specific to mesothelioma. In addition, state cancer registries, which provide limited insight into mesothelioma case numbers, do not routinely collect information about an individual’s occupation or exposure history [30]. Therefore, as part of a larger feasibility study for establishing a national mesothelioma registry, a two-year project was undertaken to develop and pilot test data collection tools using a local listing of mesothelioma patients from a university-based mesothelioma research and treatment center.

## 2. Materials and Methods

For this effort, the project team included a group of nationally recognized thoracic surgery clinicians who care for mesothelioma patients, clinicians with expertise in the prevention and care of high-risk occupational cohorts, and registry design and management experts. This team worked collaboratively to perform the following:

- Establish an Advisory Board to guide the local registry design process and provide feedback on the data collection tools.
- Create electronic data collection tools, including:
  (1) A patient exposure questionnaire;
  (2) A clinical data collection tool to capture key details related to a patient’s mesothelioma disease course.
- Revise the data collection tools based on feedback obtained from a series of key informant interviews.

### 2.1. Establishment of a Mesothelioma Registry Advisory Board

An Advisory Board was created to provide input on the data collection tools and to guide the overall local registry development process. Potential members were identified in consultation with representatives from a mesothelioma research advocacy organization and members of the scientific and clinical mesothelioma community. Ultimately the Advisory Board consisted of 10 persons, including mesothelioma clinicians (physicians and nurses); epidemiologists; and registry experts from five different countries, including Australia, Canada, Italy, the United Kingdom, and the United States. Invitees were asked to serve on the Advisory Board for at least two years and to participate in one-to-two meetings per year held in a hybrid format both in person (prior to the COVID-19 pandemic that began in 2020) and on a web-based platform.

### 2.2. Development of Data Collection Tools

The project team first reviewed the literature to draft a paper-based patient exposure questionnaire. Recognizing that there are many occupational [2,3,4,5,6,7,8,9,10], and non-occupational exposure sources [12,13,14], including para-occupational and environmental sources known to cause mesothelioma, specific activities and exposure opportunities of interest were identified. As the risk of developing mesothelioma after asbestos exposure has been shown to be dose-related [31], questions to capture the intensity, frequency, and duration of exposure were then considered for inclusion. Additionally, given the average latency period is approximately 30 years between asbestos exposure and development of mesothelioma [32], questions to collect details on the timing of exposure were included. In drafting this tool, the team reviewed and adapted questions from several validated patient surveys, such as the “ReNaM” questionnaire that was created by the Italian National Institute for Occupational Safety and Prevention for use in their National Mesothelioma Register. Working with mesothelioma treatment and research clinicians who already collect key clinical data elements for other mesothelioma research efforts, the team also drafted a clinical data collection tool to capture key details from the patient’s medical record related to the patient’s disease course and treatment.

Recognizing that shorter data collection tools would likely enhance participation, but that sufficient detail must be collected to meet the goals of the registry, the Advisory Board was asked to review each item included on the data collection tools to determine if the information was (1) needed for registry purposes, (2) “nice to know” but not necessary, or (3) not needed for registry purposes. In addition, the Advisory Board was tasked with identifying data gaps and determining if additional questions needed to be included.

Electronic versions of the patient questionnaire and clinical data collection tool were built using Research Electronic Data Capture (REDCap) software. REDCap is a secure, web-based, *Health Insurance Portability and Accountability Act (HIPAA) Privacy Rule* compliant application for building and managing on-line surveys and databases [33,34]. Before and after the creation of the electronic data collection tools, a series of structured key informant interviews with mesothelioma patients were also conducted to obtain feedback on the patient questionnaire. Institutional Review Board approval was obtained from the University of Maryland’s Human Research Protection Office.

### 2.3. Process for Conducting Key Informant Interviews

To participate in the key informant interviews, patients had to have a diagnosis of malignant mesothelioma, be able to provide informed consent, and be willing/able to complete a questionnaire and participate in an interview. Patients were excluded from participation if they were unable to provide consent or were diagnosed with a malignancy other than mesothelioma. In total, 18 mesothelioma patients were identified as potential participants, but only 16 were approached by the thoracic surgery clinical staff during their routine clinic or follow-up appointments to determine their interest in participating in this project. The other two patients were deemed not eligible due to their current state of health. Consented participants were asked to (1) complete either a paper or an electronic version of the exposure questionnaire in the presence of a study team member, thus allowing for questions and comments to be made and documented during completion of the survey, and (2) participate in a structured interview to provide feedback on the overall length of the survey, clarity of questions and response options, and ease of completing the questionnaire. Key informant interviews for those who completed the paper-based version of the questionnaire were conducted in-person; however, due in part to the COVID-19 pandemic and to allow for enrollment of patients receiving care elsewhere, all study activities for those who completed the electronic questionnaire were completed remotely (e.g., over the phone).

After receiving feedback, the study team reviewed the questionnaire responses, looking for any inconsistencies or unexpected answers, and asked participants probing questions to solicit clarifying information as needed. All patients who participated in the key informant interviews were also given the opportunity to have their data included in a local mesothelioma patient registry.

### 2.4. Process for Pilot Testing the Clinical Data Collection Tool

To obtain feedback on the clinical data collection tool, the clinicians and researchers responsible for abstracting data from the medical record for consented patients were asked to comment on the ease of use, content collected, and time requirements for completion. They were also instructed to comment on the overall patient enrollment process, and any difficulties encountered, and provide recommendations for improving the implementation processes. Results from interviews with the clinicians and key informant were shared with the Advisory Board and led to further refinement of the data collection tools.

### 2.5. Protocols for Minimizing Potential Risks for Participants

To minimize potential risks, namely the loss of privacy or breach of confidentiality, all paper records containing identifiable information are kept in secure offices within locked cabinets. All electronic data are stored in password-protected files on a secure server and accessible to only a limited number of authorized research team members. A unique study identification (ID) number has been assigned to replace patient names and any identifiable information, such as residential address information, captured for long-term registry use, and it will be available only to a limited number of authorized research team members who need the information to answer a specific research question under an IRB-approved protocol.

## 3. Results

### 3.1. Early Feedback

Feedback obtained early in the development process, from members of the Advisory Board and others in the mesothelioma community, suggested that the litigious climate in the U.S. surrounding asbestos-related disease arising from workplace exposures could be a deterrent to patient participation. This concern is related to the risk of participant information being subpoenaed and used in a court of law [35]. Therefore, in addition to the safeguards typically applied to maintain patient confidentiality, such as assigning unique subject identification codes, password-protected files, secure servers, and limited access to data, a Certificate of Confidentiality from the U.S. National Institutes of Health (NIH) was obtained. This Certificate provides an additional level of assurance as it precludes researchers from disclosing any information, documents, and/or biospecimens collected for research purposes that can identify an individual in any “federal, state, or local civil, criminal, administrative, legislative, or other action, suit, or proceeding, or as evidence, even if there is a court subpoena” unless the individual gives permission [36]. Language regarding the Certificate of Confidentiality was included in the study consent documents and discussed with all potential participants.

### 3.2. Conduct of Key Informant Interviews and Contents of Final Data Collection Tools

The final version of the patient questionnaire included detailed questions about the patient’s medical, occupational, and residential history. For example, as part of the occupational history, patients were asked, using several open-ended questions, to list all jobs they have held, starting with earliest and including years of employment, description of the work, and machinery and materials involved. Although some participants asked if only “relevant” jobs should be listed, the questionnaire instructed participants to include all jobs held to avoid missing potential exposures perhaps not known to the patient or not yet identified as a potential hazard. The questionnaire also presented participants with a listing of specific high-risk industries (e.g., construction, ship building or maintenance, firefighting, etc.) and materials that potentially contained asbestos (e.g., automotive brake or clutch linings, fireproofing materials, etc.). For each of the items listed, participants were asked to identify if they worked in the industry or with the material identified. If they responded affirmatively, they were then asked to identify the earliest year and the total number of years they either worked in the industry or with the material. In addition, information about specific environmental exposures (e.g., living within three miles of a shipyard or other potentially high-risk industry), hobbies (e.g., home renovation, automobile work), and other extra-curricular activities was collected to help identify the potential source of their asbestos exposure (Table 1). 

Participants accessed the final version of the electronic questionnaire using a secure weblink and passcode. Information entered into the web-based questionnaire was stored locally on a secure server and linked electronically to clinical data extracted from the medical record by study team clinicians. As shown in Table 2, key clinical data elements collected included vital status information and details related to the patient’s mesothelioma diagnosis, as well as procedures, treatments, and surgeries performed.

### 3.3. Findings from Key Informant Patient Interviews

In total, 18 mesothelioma patients were identified as potential key informants; however, based on advice from their clinicians, two patients were not asked to participate as they were newly diagnosed and “*emotionally devastated*.” Two patients who agreed initially, later declined. One of these patients stated that they “*felt overwhelmed*” with their medical care and the other, who had pending litigation related to their mesothelioma diagnosis, ultimately declined after speaking with their lawyer. Additionally, three individuals reported that they did not have internet access at home, precluding them from being able to provide feedback on the electronic version of the questionnaire. Ultimately, interviews with three key informants were conducted to obtain feedback on the paper version of the questionnaire and interviews with an additional eight key informants were conducted to solicit feedback on the electronic version.

#### 3.3.1. Select Characteristics of the Key Informants

Combining data from both sets of key informant interviews, feedback was obtained from a total of 11 mesothelioma patients, nine men and two women. All participants were white with a mean age of 63 ± 6 years and were diagnosed with pleural mesothelioma.

Of the individuals who provided feedback on the web-based questionnaire, three reported that their mesothelioma was likely related to work exposures, two from military exposures, and three from other exposures (e.g., paternal occupational exposure). The question soliciting this information was added to the survey after two of the three individuals who provided feedback during the initial round of key informant interviews suggested including an open-ended question to allow participants to comment on what they think is the source of their mesothelioma.

Most participants who provided feedback on the web-based questionnaire reported that they considered themselves “fairly computer savvy for their age,” although two asked a family member to assist them with entering responses into the on-line form.

#### 3.3.2. Patient Acceptability

Overall, comments received on both the paper and web-based version of the questionnaire were positive. All of the participants reported that the length of the survey was “appropriate.” Participants commented that the questionnaire was “*thorough, but not too exhausting*,” “*seemed to ask all the right questions, drilling down at times for more information, but not requesting exhausting detail*,” and “*can’t be shorter and still capture enough useful details*.” Comments from those who completed the web-based version of the questionnaire included that it was “*easy to access and navigate*”, and that the overall flow of the questions was logical. Although not specifically asked, some participants offered that they had been through the litigation process and therefore found this survey much easier to complete as they had already answered similar questions.

Individuals who completed the paper version of the questionnaire did so within 20 min. However, on average, it took approximately 45 min, with a range of 15 min to 2 h, for participants to complete the web-based questionnaire. The wide variability in completion time was largely dependent on two factors: (a) the participant’s typing ability and (b) the extent of their work history. Despite the amount of time it took to complete the survey, no recommendations were made for deleting specific questions.

The three sections of the questionnaire that took the longest for participants to complete included the work history, residential history, and current medication use. As this was identified in the initial round of key informant interviews, during subsequent scheduling of study visits participants were encouraged to have documents or items on hand that may help their ability to recall information (e.g., old resumes, medications, or lists etc.). Those who had such materials available during the interview reported that they felt it significantly decreased the amount of time they needed to complete the survey but remembering specific dates of employment and residence still posed challenges for some. For example, one participant noted that individuals who moved frequently due to military obligations may not be able to recall all places of residence. Furthermore, one individual who worked as a laborer, through a temporary employment agency, had difficulty answering questions related to his work history as his work location and job tasks varied week to week. Despite these difficulties, all the participants completed the survey without skipping questions.

Suggestions for improving the patient questionnaire mostly focused on defining specific terms, such “asbestos surveillance program,” “pleural plaques,” and “carbon nanotubes,” or adding clarifying instructions to a stem of a question. For example, in the work history section some informants wanted to know if they should start with their most recent job or earliest job, and if all jobs had to be listed, or only jobs they thought were pertinent. When asked if questions should be added, two participants suggested adding more questions related to the use of personal protective equipment (PPE) to capture not only whether they used PPE, but also what type of PPE they used at work (e.g., respirator, gloves, face shield, etc.) and how they used it.

All patients who participated in the key informant interviews also consented to having their data retained for inclusion in a mesothelioma patient registry established locally.

### 3.4. Clinic Staff Acceptability

Overall, clinic and research staff reported the amount of time needed to complete the data abstraction for the clinical data collection tool was “reasonable,” ranging from five to 20 min per patient. It was reported that the length of time required to complete the tool for each patient varied, in part, according to the staff person’s level of familiarity with the patient’s care. It was also noted that specialty clinics, where patients with this orphan disease are often referred to for care, already collect much of the requested clinical information for other research efforts, but that it would be helpful to have a single designated clinical research staff team member responsible for abstracting data from the medical records. Additionally, it was noted that, as with all research activities, all data should also be reviewed by a second person since data abstraction and data entry errors can occur. One concern that was raised involved the challenge of keeping the clinical information up to date, but it was thought that this would become easier as data collection becomes more routine.

## 4. Discussion

The lack of a national mesothelioma registry that combines exposure and clinical data has hampered epidemiologic efforts to better characterize exposure circumstances and other risk factors associated with disease incidence and mortality within the U.S. It has also made it difficult to identify participants who may be eligible to participate in and benefit from clinical trials. However, several lessons learned from this project can help inform the development of such a registry.

First, as suggested by others, the unique legal environment in the U.S. may deter some individuals from participating in a mesothelioma patient registry, especially if detailed exposure and work history information is collected. Specifically, the concern is that if registry data differed from information collected for a legal purpose, it could be used to support the employer’s defense in a compensation claim, thereby unintentionally harming the participant [35]. As a result, registry personnel must provide assurance to participants that all possible precautions are being implemented to maintain their confidentiality. In this U.S. context, obtaining a Certificate of Confidentiality from the NIH is one way to do this, as it provides an extra layer of protection for maintaining confidentiality by legally prohibiting disclosure of personally identifiable or sensitive information even when subpoenaed [36]. Other proposed strategies for minimizing risks have included (a) not collecting information that would be used in compensation cases, (b) removing all personal identifiers from the data, and (c) holding all data that could be used in compensation cases in a format that would not allow access until the case is closed [35]. However, each of these proposed options would limit, to a varying degree, the potential utility of a registry.

A second valuable lesson learned was that timing of recruitment matters within this population. While early identification of patients is ideal given the aggressiveness of the disease, approaching patients early after receiving their diagnosis to collect detailed exposure information via questionnaire may cause undue stress and result in the patient declining to participate. Coordinating and communicating with the patient’s clinician may help registry staff in identifying a more appropriate time to approach the individual. However, waiting to approach a patient to collect information may also be challenging, given the rapid progression of the disease. Although not ideal, another alternative for data collection, if the patient is too ill to complete a questionnaire, could be to collect information from the next of kin as is done with other registries [35,37].

A third lesson gained from this project is that collection of detailed exposure and clinical data for this patient population is feasible using on-line data collection tools; however, to permit increased participation, other methods for completing questionnaires must be made available for those who are not comfortable completing the on-line survey themselves or who lack internet access, especially given the older age of this population. In 2012, a review of the literature suggested that only 34% of those over the age of 75 had home internet access [38]. However, it was also noted that internet use was growing the fastest among older age groups worldwide [38]; therefore, the percentage of older adults with internet access at home is likely to be much higher now, a decade later. Regardless, allowing patients the option to participate and complete a questionnaire via an interview with registry staff, although potentially more resource intensive, must remain an option.

Finally, to ensure sustainability of registry efforts, there must be dedicated resources to allow for updating/maintenance of the electronic database and infrastructure, and to provide adequate staffing. With adequate resources, the collection of exposure information from the patient, as well as abstraction of clinical data from medical records, can be completed by dedicated registry research staff. Involvement of clinical staff in specialty clinics, who are currently collecting clinical data for research purposes among this patient population and are familiar with the individual’s care, can help reduce the amount of time needed to capture the clinical data. However, to help ease the burden of data collection efforts and to improve consistency of data across other sources, linkages to other data systems, such as the National Vital Statistics System and state-based cancer registries should be explored. Additionally, to permit work history responses to be easily coded according to standard occupational codes for analyses, options for collecting patient responses using a more standardized approach should be considered. One such option, which is currently under investigation for use among mesothelioma patients, is to use the Occupational Data for Health (ODH) modules created by U.S. National Institute for Occupational Safety and Health (NIOSH) researchers. These ODH modules suggest using standard data elements, structure, and vocabulary in health information technology systems to collect self-reported data related to one’s “usual” work, past and present jobs, employment, and retirement status, including deployments to combat zones [39].

### Strengths and Limitations

The effort described here provides insight into the feasibility of establishing a national mesothelioma patient registry in the U.S. that collects both exposure and clinical data. The use of key informant interviews provided detailed patient feedback on the data collection tools and the lessons learned will help inform the development of a next iteration of the patient history. However, one limitation is the potential for selection bias. While it is possible that individuals who were approached but did not participate in the key informant interviews may have provided different feedback on the questionnaire compared to those who participated, their lack of participation is unlikely to have had a significant impact on the overall lessons learned. Another limitation is that the key informants, while representative of the referral clinic’s mesothelioma patient population, were on average younger than the average age of mesothelioma patients at time of diagnoses in the U.S. (63 years of age versus 72) [40]. While this may limit the generalizability of the findings, ongoing work is currently underway to further evaluate the use of these data collection tools in a larger group of mesothelioma patients.

## 5. Conclusions

A national mesothelioma registry can help advance the scientific knowledge and understanding of the exposure circumstances and clinical history, disease progression, and treatment course of mesothelioma patients in the U.S. Key informant interviews suggest that capturing such information using on-line data collection tools is feasible; however, given the unique characteristics of this patient population and the U.S. legal environment, specific concerns related to data confidentiality, timing of enrollment, and availability of resources must be considered. These lessons learned, combined with additional pilot testing of the data collection tools among a more diverse group of mesothelioma patients, will help inform the development of a national mesothelioma patient registry, as well as other cancer registry development efforts, in the U.S.

## Figures and Tables

**Table 1:** Data Elements Captured on Exposure Questionnaire.

Examples of Specific Elements	
Demographics	Date of birth, race, sex, education, marital status, etc.
Medical History	History of certain medical conditions (e.g., hypertension, diabetes, endometriosis, etc.) History of pleural effusion, pleural plaques, or pulmonary fibrosis Cancer history Family cancer history Current medications Allergies
Occupational History	Employment status and work history Enrollment in asbestos surveillance program at work Involvement with specific exposure activities (e.g., firefighting, manufacturing of ceramics, brake repair, etc.) Use of personal protective equipment Military history
Residential History	Lived in close proximity to specific industries (e.g., shipyards, quarries, railway yards, waste disposal sites, etc.) Lived or worked abroad Known or suspected presence of asbestos at home Locations where lived for more than two years
Occupational history of potentially exposed household member	Lived with someone exposed to asbestos Occupational history of asbestos-exposed individuals At risk behaviors (handling contaminated clothes, etc.)
Hobbies and extra-curricular activities	Home repairs/renovations Motor vehicle work (brakes) Use of specific items (i.e., talcum powder, etc.)
Additional exposure information	Smoking history Patients’ thoughts on source believed to cause their mesothelioma

**Table 2:** Data Elements Captured on Clinical Tool.

Examples of Specific Elements	
Initial Visit Information	Eastern Conference Oncology Group (ECOG) Score Initial diagnosis Biopsy date Initial pathology
Procedures Performed	Endobronchial ultrasound (EBUS) details Laparoscopy details Mediastinoscopy details Video-assisted thoracoscopic surgery (VATS) details
Pleural Mesothelioma Surgery/Peritoneal Surgery Details	Date of surgery Surgery type Intraoperative procedures and adjuvant treatments Surgical biopsy results Lymph node involvement Peritoneal cancer index (PCI) score TNM staging (tumor extent, lymph node involvement, metastasis) Death within 30 days Discharge status
Neoadjuvant Chemotherapy/Systemic Therapy	Treatment date Type of treatment Treatment response
Clinical Trial Information	Trial involvement Collection of biospecimens
Recurrence details	Recurrence date and location
Vital status information	Date of last follow-up Current status (alive with disease, no evidence of disease, dead of disease, lost to follow-up)

## Data Availability

Data sharing not applicable.
